# Postmortem Analysis Using Different Sensors and Technologies on Aramid Composites Samples after Ballistic Impact

**DOI:** 10.3390/s20102853

**Published:** 2020-05-17

**Authors:** Ignacio Rubio, Antonio Díaz-Álvarez, Richard Bernier, Alexis Rusinek, Jose Antonio Loya, Maria Henar Miguelez, Marcos Rodríguez-Millán

**Affiliations:** 1 Department of Mechanical Engineering, University Carlos III of Madrid, Avda. de la Universidad 30, 28911 Leganés, Madrid, Spain; andiaza@ing.uc3m.es (A.D.-Á.); alexis.rusinek@univ-lorraine.fr (A.R.); mhmiguel@ing.uc3m.es (M.H.M.); mrmillan@ing.uc3m.es (M.R.-M.); 2 Laboratory of Microstructure Studies and Mechanics of Materials (LEM3), Lorraine University, 7 rue Félix Savart, BP 15082, 57073 CEDEX 03 Metz, France; richard.bernier@univ-lorraine.fr; 3 Department of Continuum Mechanics and Structural Analysis, University Carlos III of Madrid, Avda. de la Universidad 30, 28911 Leganés, Madrid, Spain; jloya@ing.uc3m.es

**Keywords:** piezoelectric sensor, CT tomography, 3D scanner, damage, composite

## Abstract

This work focuses on the combination of two complementary non-destructive techniques to analyse the final deformation and internal damage induced in aramid composite plates subjected to ballistic impact. The first analysis device, a 3D scanner, allows digitalising the surface of the tested specimen. Comparing with the initial geometry, the permanent residual deformation (PBFD) can be obtained according to the impact characteristics. This is a significant parameter in armours and shielding design. The second inspection technique is based on computed tomography (CT). It allows analysing the internal state of the impacted sample, being able to detect possible delamination and fibre failure through the specimen thickness. The proposed methodology has been validated with two projectile geometries at different impact velocities, being the reaction force history on the specimen determined with piezoelectric sensors. Different loading states and induced damages were observed according to the projectile type and impact velocity. In order to validate the use of the 3D scanner, a correlation between impact velocity and damage induced in terms of permanent back face deformation has been realised for both projectiles studied. In addition, a comparison of the results obtained through this measurement method and those obtained in similar works, has been performed in the same range of impact energy. The results showed that CT is needed to analyse the internal damage of the aramid sample; however, this is a highly expensive and time-consuming method. The use of 3D scanner and piezoelectric sensors is perfectly complementary with CT and could be relevant to develop numerical models or design armours.

## 1. Introduction

The current combat helmets are made of polymeric material reinforced with high ballistic resistance fibres, as aramid and high molecular density polyethene fibres. The main feature of the helmet is to minimise the brain injury that may affect the wearer due to blast or ballistic impact. Concerning the ballistic threats classification, combat helmets could be divided into perforating and non-perforating threats. Perforating threats are presented in war scenarios such as metalcore ammunition and long-range bullets [1]. These projectiles require tough materials to stop and break the bullet, as in the case of ceramic in bulletproof vests [2,3]. Non-perforating projectiles are generally made of lead core. This ammunition has a high stopping power due to the high energy transmission and is usually used for police equipment.

In this case of non-perforating ammunition impact on a combat helmet, the higher part of the kinetic energy of the projectile is absorbed into delamination of the helmet, causing less non-penetrating deformation in the human body. Therefore, the damage extension is one of the most influential parameters for designers and is important for testing and evaluation agencies [4].

Damage analysis has been widely analysed in composite materials such as carbon or glass fibres [5,6,7,8,9,10]. Main damage analysis techniques are based on non-destructive ultrasonic inspection. C-SCAN provides a projection of the damage extension presented in all the plies but is not possible to distinguish the damage type (matrix cracking, matrix crushing or fibre failure) and the location at the specimen thickness. Segreto et al. [9] analysed complete applications of these NDT (Non destructive test) techniques to CFRP (Carbon fibre reinforced plastic) composites subjected to low-velocity impact. Wu et al. [10] complemented the use of C-Scan with microscopic analyses in his study of 5D hybrid composites subjected to low-velocity impact. Due to this limitation, C-Scan and phased ultrasonic array systems are commonly used to analyse the location of the delamination through the thickness of the laminate. 

Micro-tomography is a relatively novel technique with high potential for detection of defects and damage in a wide variety of materials. In composite material with non-appreciable defects in a visual inspection, the use of micro-tomography makes it possible to detect and place on 3D the presence of defects with reasonable accuracy, placing it above other inspection techniques such as ultrasonic. Vasudavan et al. [11] studied the stacking sequence of hybrid composites influence subjected to low-velocity impact on damage, obtaining more accurate results than those obtained with C-Scan or SEM. Fidan et al. [12] compared the response of glass and aramid/glass composite under low-velocity impact in terms of damaged induced on plates. However, these techniques have not been sufficiently developed in aramid fibre composites due to their high absorption and signal attenuation. 

Aramid plates have been usually studied by merely visual inspection [13] or by cutting transversely through the impact zone to evaluate the failure mechanisms [14,15,16,17,18,19]. or One of the first studies was developed by Zhu et al. [16] in a wide range of strain rates. Van Hoof [17] carried out damage inspection of the compound in a similar way. Glossop et al. [20] developed a method based on image-enhanced backlighting applicable to translucent composite materials such as Kevlar/epoxy and glass/epoxy to detect delamination with high resolution. Slwasky et al. [21] studied aramid and Carbon composite response against different nose shape projectiles impacted at the same impact energy. The damage induced on plates was analysed through microscopic and by visual inspection. Tan et al. [22] conducted the post-test evaluation of the damaged helmets using visual observation, optical microscopy and computed tomography scan. Palta et al. [23] performed a visual inspection of the damage in the combat helmet in case of a rifle impact and numerical analysis of the different failure modes: fibre breakage, matrix cracking, compressive matrix damage, and delamination. Recently, I. Rubio et al. [24] developed a damage analysis of combat helmets submitted to several projectile perforating impacts by computed tomography.

In this work, a new methodology based on different complementary sensors to measure the damage in the aramid fibre composite is presented. Three devices have been used for this purpose: pressure sensors to measure the energy absorbed by the sample during impact, 3D surface scanner and computed tomography. This procedure allows obtaining the total energy absorbed by impacted aramid samples, and the internal and surface damage for possible control by the evaluation agencies according to different projectile geometry and impact velocity. The damage resulting from tests with two different impactors will be analysed to show the effectiveness of the method. The critical gap that leads this work is the need for non-destructive techniques, such as micro-tomography, for aramid composites, since the damage inspection is mainly based on the destructive test. 3D Scan, rarely used in this field up to date, also allows comparing a damaged sample with an undamaged plate to observe, deformations and defects.

After reviewing the literature that the authors have been able to access, such a detailed study of postmortem damage in aramid has not been found. Here, a new methodology has been proposed that combines the use of micro-tomography, which has been seen to be increasingly used in the detection of internal failures in composite material since its medical conception, and the 3D scanner that allows obtaining digital models of the specimen failure and can compare them with other models or specimens without damaging. On the other hand, the use of force sensors to quantify the temporal evolution of the resisting force during the impact process allows mechanical parameters, such as the force itself, deflection or stiffness, to be related to the presence of material failures.

This methodology is extensible to other personal protection elements such as combat helmets and not only to quantify the local failure around the impact zone but also to analyse global deformations in the helmet and its subsequent influence on its ballistic performance under later threats.

## 2. Materials and Methods

### 2.1. Material and Structure: Aramid Composite Plates

Aramid fibres are a class of resistant and thermostable synthetic fibres. They are widely used in aerospace and military applications, fabrics for bulletproof vests and ballistic composites, bicycle pneumatic tyres and as a substitute for asbestos. The chain molecules of aramid fibres are mostly oriented along the axis of the fibres, which makes it possible to benefit from this chemical bond strength. Among others, these composites present high mechanical strength/weight ratio, structural stiffness (Young’s high modulus and low elongation at break), high tenacity, high cut resistance and low thermal conductivity.

Aramid composite plates have been manufactured through hand-made stacking of single plain wave woven fabrics of K129 aramid fibres, embedded in Polyvinyl Butyral Phenolic matrix (PVB) and then using hot-pressing; subsequently, the composite was cured. This thermoforming technique allows obtaining a low resin content on composite, close to 18%. Thanks to this procedure, a lightweight composite combined with high strength due to high fibre content (approximately 82%) has been obtained. Specimen test was squared 130 × 130 mm^2^ with an areal density (units typically used in personal protection) of 8.86 kg/m^2^. This material has been used in different analyses in other works [18,24,25,26,27,28].

**Figure 1:**
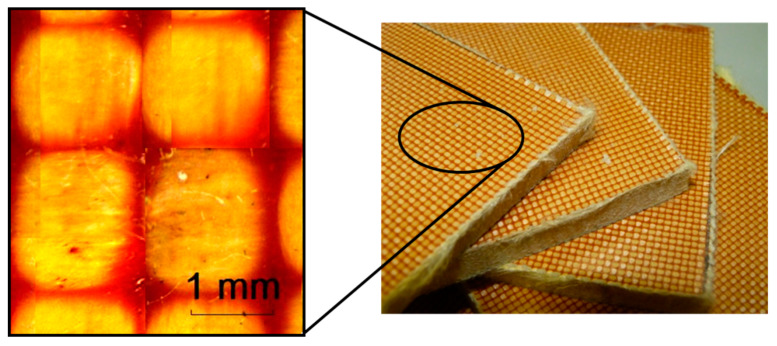
K129 aramid composite used in this work and description of the size of the fibres.

Aramid composite material used in this work is shown in Figure 1. The scale presented gives an idea of the size of the fibre in the compound and leads to a more macroscopic analysis of the induced damage.

As it is mentioned above (Section 1), aramid fibres are typically used in the defence sector for personal protection and this reason necessitates a high confidentiality around the mechanical properties, fibre and fabric geometry of these composites (i.e., through-thickness dimension). Some known properties of aramid K129 fibres [29] are presented in Table 1

### 2.2. Ballistic Impact Device: Laser and Piezoelectric Sensors

**Figure 2:**
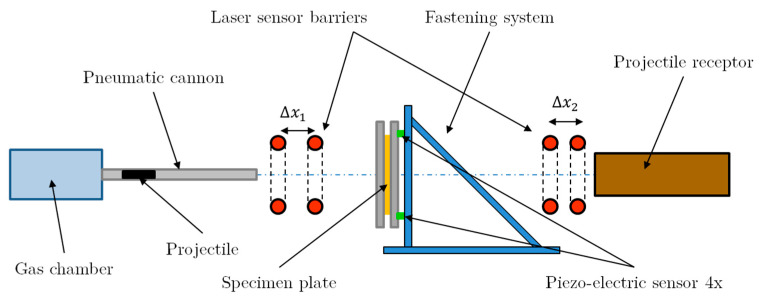
Experimental impact setup description.

The experimental setup has been used in several ballistic studies with different materials [30,31,32,33]. The main device has been designed in LEM3 (Laboratory of Microstructure Studies and Mechanics of Materials) and is based on a 13 mm barrel diameter pneumatic launcher, Figure 2. Once the projectile is ejected from the muzzle, the impact velocity of the projectile is measured by two laser-gate sensors separated 50 mm. Measuring the time interval that the projectile takes to travel that distance, the impact velocity is determined by the expression, ΔX/Δt. It has been verified that the maximum error on impact velocity is approximately ±1 m/s [30,31,32,33]. In the case of specimen perforation, another similar laser barrier separated 5 mm system measures the residual projectile velocity.

The plate fixation device is a rigid structure that allows the secure placement of the specimen. Two steel frames fasten the sample in the perimeter, allowing an effective impact area of 100 × 100 mm^2^. This structure is close enough to the cannon muzzle to ensure the normality of the impact and placed in the centre of the plate.

**Figure 3:**
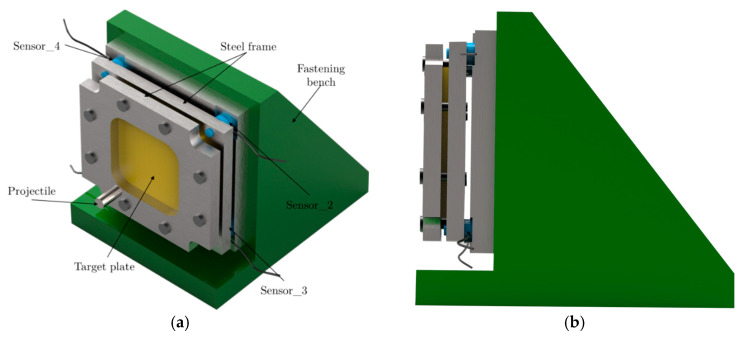
Target elements locations. (**a**) Perspective view; (**b**) Lateral view.

Four piezoelectric force sensors (9011A Kistler) are used to measure the global force induced by the projectile on the target during impact. These sensors are fixed over the plate holder (Figure 3) and allow measuring the dynamic force along the impact velocity direction. The range of the maximum force that the sensors system can measure is 60–80 kN (15 ± 5 kN), and the natural frequency is 65 kHz for each sensor. The four sensors are coupled to an amplificatory device.

The structural support has been designed by Kistler and presents a high stiffness and yield stress ($\sigma_{y}\approx \;$850 MPa) to avoid bending during an impact event. The mass of the complete structure is close to 40 kg (Figure 3).

**Figure 4:**
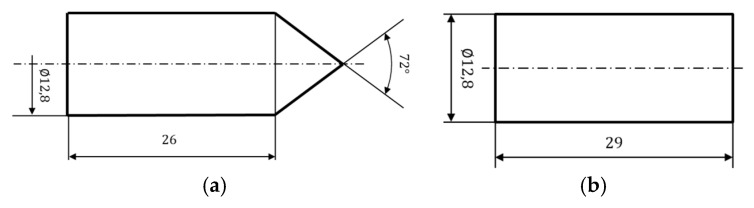
Dimensions of the projectiles for impact. (**a**) Conical projectile; (**b**) Blunt projectile. Dimensions in mm.

To compare the results obtained with similar impact energies, two types of rigid projectiles of 28grams mass and different geometries (conical and blunt-nosed shape) have been manufactured of maraging steel with heat treatment to increase their hardness close to 600 HV. Therefore, the local and global failure modes in the aramid composite plate depend on the projectile nose. The diameter of the projectile is 12.8 mm which is slightly smaller than the diameter of the barrel, to avoid the use of the sabot. The dimensions of the two projectiles are shown in Figure 4.

### 2.3. Postmortem Analysis: CT-Scan (Computerised Tomography Tomography and 3D-Scanner)

#### 2.3.1. CT-Scan

**Figure 5:**
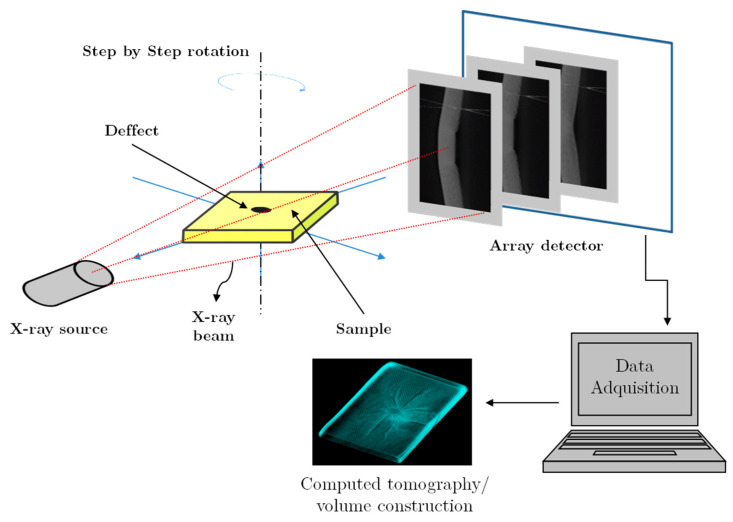
Computerised micro-tomography (CT) process scheme for measurement and acquisition.

Computerised micro-tomography (CT-scan or CT) is a powerful technique which consists of X-Ray scanning axial images of high quality and precision. An X-ray beam is projected on the sample to be analysed, and part of the radiation that has not been absorbed by the object is collected by detectors like a spectrum. A data acquisition system transforms the data obtained by the sensors into digital images. From this, a grayscale voxel image that depends on the number of electrons that has been absorbed by the object is obtained. Greyscale is limited by white colour, associated with undamaged material, and black colour, due to a change in density because of the presence of air as a result of breaks or defects. A scheme of a CT process is shown in Figure 5.

In this work, the internal damage of aramid plates after impact has been analysed using the CT-scan technique. Images were obtained by an X-ray micro-tomography device (model EasyTom Nano, Rx Solutions) with a minimum X-ray beam power of 160 kW which is possible to scan with a precision of 50 μm. Each plate takes around 4 h of scanning, requiring later image post-processing (AVIZO and X-Act 2.0 software was used). Tomography on aramid composites is a technique that has been rarely used, so it is a promising process of damage measurement with a wide range of applications in this material [22,24]. Some representative parameters used to scan the samples are presented in Table 2.

#### 2.3.2. 3D-Scanner

The surface damage and the local and global deformation measurements of the tested plate have been obtained using an HP brand scanner (model HP 3D Structured Light Scanner Pro S3, scanning volume 60–500 mm^3^ and up to 0.05 mm resolution). The minimum scan time for single image capture is 2 s. The equipment has two HP high definition cameras for stereoscopic image capturing combined with a structured light projector (ACER, model K132). It projects a beam of black/white line patterns on the object (structured light) with different orientations. The object to scan is positioned on an automatic 360° rotating platform while the cameras capture the light reflected on the object. The images of the object taken during the process need to be aligned for the final shape representation.

**Figure 6:**
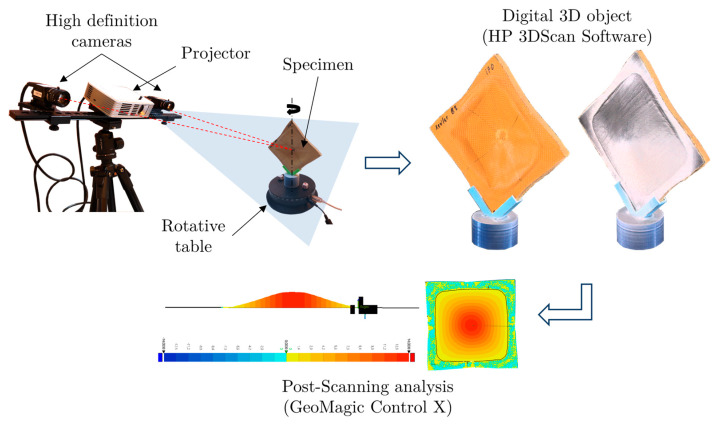
3D scanner equipment to measure permanent deformation on samples.

As part of the post-processing of the scan, final shape representation is exported to the GeoMagic Control X^TM^ software that allows the display and quantification of dimensions, superficial defects, wear and tear compared with the no tested specimen, among others. In our case, it allows us to determine the induced deflection in the plate due to the type of projectile and impact velocity. A scheme of the scanning process is represented in Figure 6.

To the best of the author’s knowledge, the application of this technique for damage analysis on impacted aramid composites has not been previously used.

## 3. Results and Discussions

Five non-perforation tests in the range of impact velocity 90 m/s < V < 174 m/s have been carried out for both projectiles in order to observe the influence of the nose type shape on induced damage on plates. From each test, the resistance force has been obtained using piezoelectric sensors in the supporting rig (described in Section 2.2), determining the resistance force history as a function of the projectile nose shape and impact velocity. Later, two non-destructive complementary techniques (3D scanner and computed tomography) have been used to analyse surface damage and internal damage of the tested plates. The particular use of CT-scanning for internal plate inspection contrast with other techniques usually carried out, as cross-section cutting, a destructive technique that induces additional damage, or non-destructive ultrasonic inspection, not easy to use due to the high signal attenuation in aramid panels in comparison with other materials, as CFRPs or GFRPs (glass fibres).

The applicability of the non-destructive proposed methodology is shown below with two plates impacted at 174 m/s with cone and blunt projectiles (454 J, impact energy) according to the cases where the maximum damage is reached, enabling the observation of the different damage modes attributed to the different projectile nose shapes.

### 3.1. Resistance Force History by Piezoelectric Sensors

Using the laser beams sensor, it is possible to quantify the impact velocity for each test, as was mentioned in Section 2.2. All the studied cases were performed at impact velocity below the ballistic limit, so complete perforation was not achieved, and there is no projectile residual velocity.

**Figure 7:**
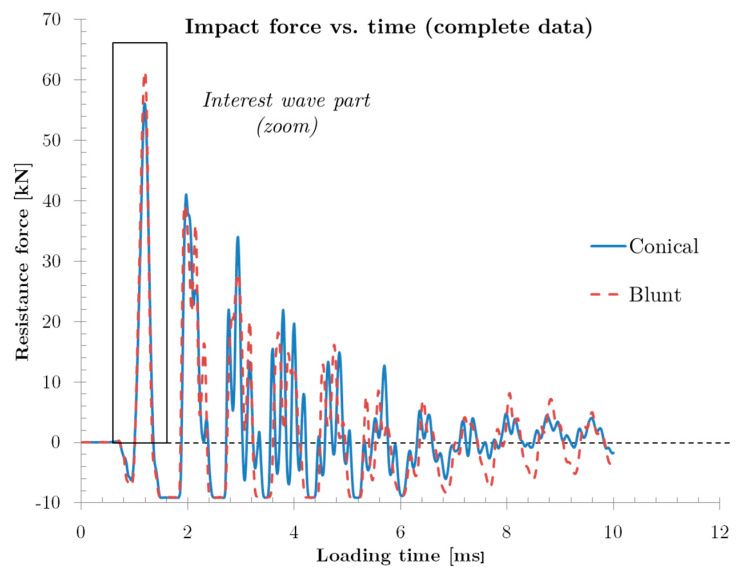
Resistance force history recorded using piezoelectric sensors for blunt and conical projectile (impact velocity v = 174 m/s).

In Figure 7 is showed the force history transmitted by the plate during the impact process and recorded using the four piezoelectric sensors for both projectiles impacting at 174 m/s. The represented wave is the sum of the individual waves of each sensor. The two curves in Figure 7 are quite similar as it is a non-perforation test using two different projectiles with the same mass. Therefore, because in these cases where the projectile does not produce complete perforation on samples, the plate absorbs most of the impact energy (around 454 J at V = 174 m/s) through failure mechanisms.

**Figure 8:**
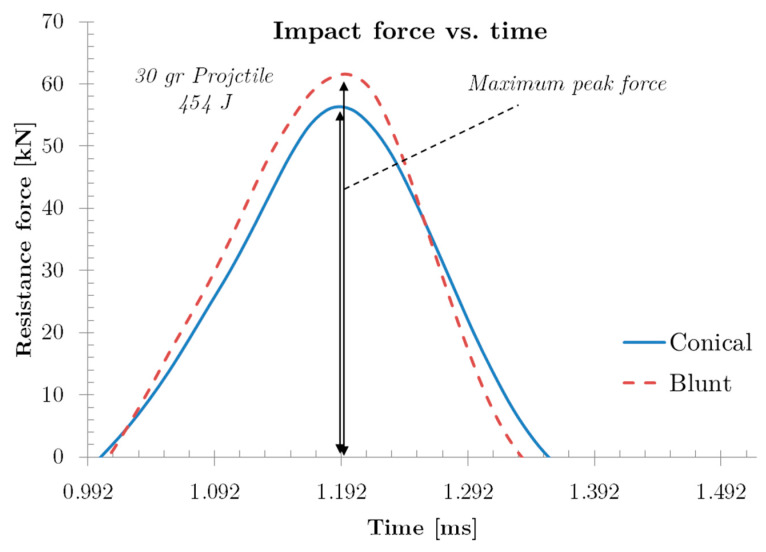
Detail of peak force obtained from resistance force history.

The most remarkable part of this figure is the first peak (shown in detail in Figure 8), corresponding to the time when the impact takes place; the rest of the curve history represents wave reflections due to little rebounds of the plates and frame on the sensors.

As mentioned, all the projectiles analysed in this work have the same mass, although the nose shape may be different. Curves represented in Figure 8 for projectiles with similar impact energy lead to different maximum impact force peak due to the different shape nose. Blunt projectile presents higher values ($F_{\mathrm{impact}}=62\;\mathrm{kN}$) than the conical one ($F_{\mathrm{impact}}=56.0\;\mathrm{kN}$). The maximum peak force occurs when projectiles transferred all kinetic energy to the plate. The time amplitude of the force-time curve of the blunt projectile (Δt=0.333 ms) is smaller than for the conical projectile (Δt=0.388 ms), this may indicate lower overall plate deformation as will be shown below.

Another interesting parameter observed in this analysis is the impulse transmitted to the plate by the projectile, which is possible to determine calculating the area under the curve of Figure 8. For the conical projectile, the impulse transmitted is 9.73 N·s and, for the blunt projectile, it is slightly higher, 9.27 N·s for the same impact energy, because less perforation occurs in the specimen with this projectile.

**Figure 9:**
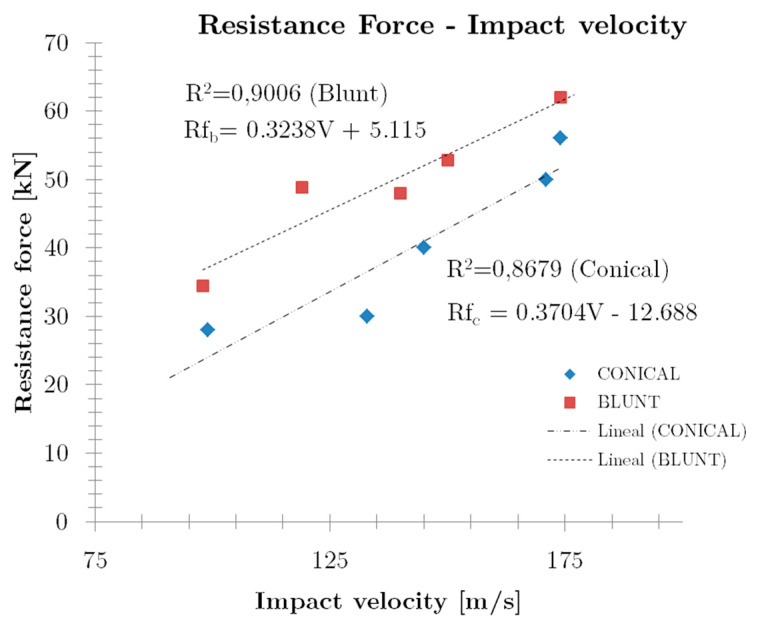
Impact velocity and resistance force comparison for both projectiles.

A correlation for the impact velocity with the maximum peak force from each test, obtained using the piezoelectric sensors, is presented in Figure 9.

The results for the impact range considered have been adjusted to a linear trend for every nose-shape. The correlation coefficients (R-value) are R^2^ = 0.9006 and R^2^ = 0.8679 for the blunt and conical case, respectively, representing a good (and adequate) correlation with experimental values, as can be seen in Figure 9. Although both slopes are quite similar, resistance force for any impact velocity is always lower in the conical case, due to its greater penetration power than the blunt projectile, reducing the stiffness of the plate and producing a lower peak force for the same impact load.

### 3.2. Failure Mechanism by CT Images

**Figure 10:**
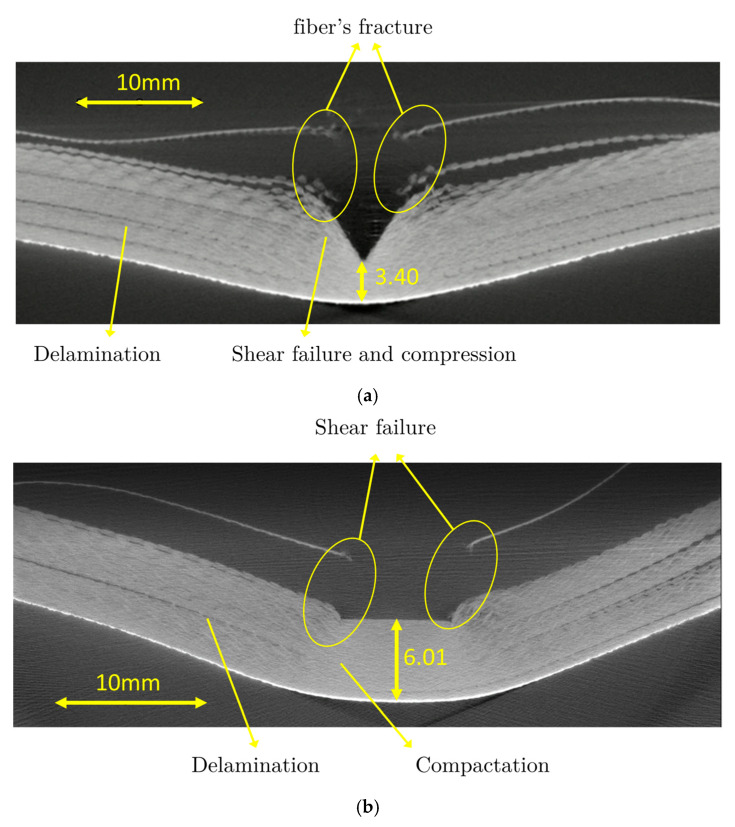
Tomography of different plates impacted to 454 J (V_o_ = 174 m/s). (**a**) Conical projectile test; (**b**) Blunt projectile test.

The failure mechanisms and the morphology of the penetration process have been analysed through computed tomography, described in Section 2.3.1. Figure 10 demonstrates the capacity of this technique to report, in a non-destructive mode, the internal damage of the specimen after impact. These images are taken close to the impact zone, where the most significant penetration of the projectiles is observed. The high-resolution images allow distinguishing delamination, ruptures and crushing.

These modes of damage are generally found in all composite materials regardless of the type of reinforcement, highlighting, according to the impact velocity, a projectile’s geometry, boundary conditions and material. Figure 10 shows the different failure modes mentioned. It is observed that the main mode of energy absorption is the delamination which is observed in other composite materials, i.e., glass [34]. Moreover, due to the impact of the different nose shape projectiles, the breakage of the fibres is found differently in the rear part for each type of specimen. 

However, the experimental tests carried out in this work in impact energy regime show that there is no complete perforation of the specimen; therefore, no fibre failure on the rear part of the composite is found. These damage modes are commonly observed and are very similar to those studied by other authors [12]. Unlike other composite materials made with glass or carbon fibre, aramid composite does not produce brittle fractures, in accordance with the mechanisms described above. To better understand the difference between CT damage analysis of a glass fibre composite and a glass fibre/aramid composite see the work developed by Fidan et al. [12]. Consequently, our images obtained by CT scanner are more similar to the glass/aramid composite.

According to Min et al. [35], the region where delamination is most widespread is the area where the impactor stopped, which is the last penetrated layer. Thus, the delamination is most notable with the conical projectile where the penetration into the specimen is higher than the blunt, Figure 10a. 

Moreover, conical projectile leads to higher layer breakage compared to the blunt-shaped projectile, Figure 10b. In the first layers at the impact zone, higher delamination is found for the conical projectile. However, in the case of the blunt projectile, fibre breakage of the first two layers is caused by pure shearing. This statement is commonly observed in the literature [18] due to the geometry of the projectile. Crushing of the fibres along the generatrix of the tip cone is caused by the conical projectile. For the blunt projectile, Figure 10b, compression and crushing of the layers in the impact zone along the thickness of the specimen is found. For the case of conical shape, a thickness reduction of 54% was observed due to penetration and compressing action of the projectile. On the other hand, as stated above, the blunt projectile does not produce a remarkable penetration, providing a reduction in the thickness of 18% due to layers compression principally. This thickness reduction generates a residual stiffness higher for the blunt projectile than the conical projectile. 

Thus, due to the morphology of the failure mechanisms described above, the conical projectile shows a better penetration capability than the blunt projectile. This observation is perfectly matched with the impact force analysis (Section 3.1) and the permanent back deformation (Section 3.3). Therefore, this technique is very useful for damage inspection in a non-destructive way due to its high quality and resolution in localised areas.

Qualitatively, this technique allows expanding the knowledge about the failure mode of composite materials, since it can be different depending on the composition of the composite under the same loading action [12].

### 3.3. Permanent Back Face Deformation by the 3D Scanner

The permanent deformation of the backside of the specimen (PBFD) after impact is measured using the 3D scanner. This technique provides the displacement field at any point of the surface of the plate. 

**Figure 11:**
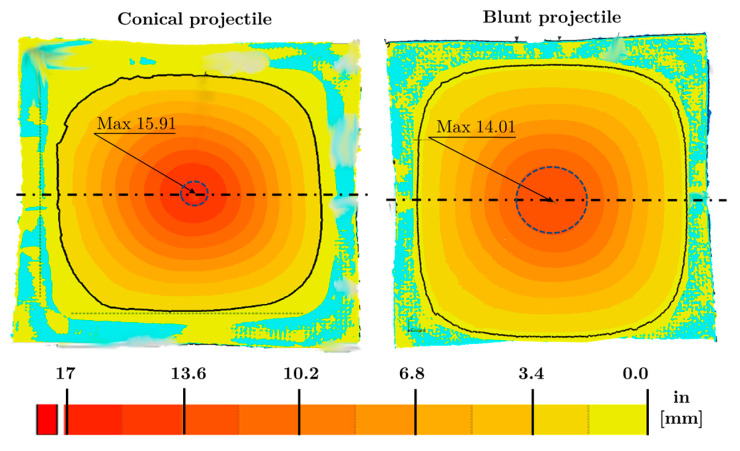
Impact direction displacement map. (**left**) Conical and (**right**) blunt projectile (V_o_ = 174 m/s).

Figure 11 shows the displacement map in the projectile impact direction on plates impacted by a conical and blunt projectile at 174 m/s. In all cases, the maximum displacement is obtained at the impact zone, close to the specimen centre, decreasing to the encastred perimeter. For the velocity presented, these maximum values are 15.91 and 14.01 mm for the conical and blunt projectiles, respectively. Due to the characteristics of the problem, symmetric patterns are displacement is achieved. Contour lines indicate higher displacement gradient for the conical tip-shape. For a particular impact velocity, the larger area of deformation is obtained with the blunt projectile (marked with the continuous black line built thanks to by the GeoMagic Control XTM software), being very close to the encastred boundary. 

**Figure 12:**
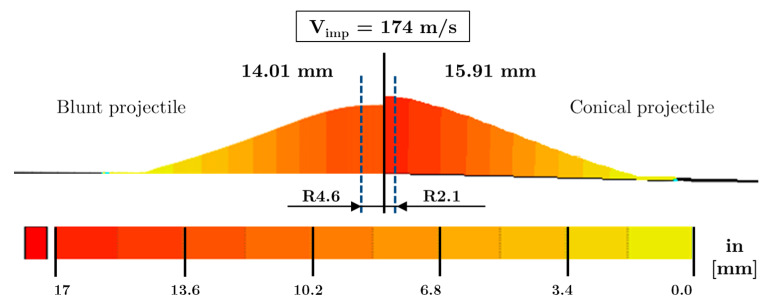
Results of the permanentback face deformation (PBFD) measured with GeoMagic Control X^TM^ from the 3D scan.

The 3D scanner allows analysing any virtual cut section of the specimen through the thickness. In particular, virtual sections by the dotted line (through thickness plane) sketched in Figure 11 are presented in Figure 12 for a better comparison. It can be observed that the specimen impacted with a blunt projectile has a lower displacement gradient and a more permanent deformation than the conical one, and it is flattened due to the morphology of the projectile, being less pronounced than with the conical projectile. The maximum deflection, reached in the conical case, is obtained strictly at the projectile/plate impact point; meanwhile, the blunt projectile presents an almost constant value close to the impact zone. 

Comparing differences in deflection due to the projectile, these are according to the internal damage observed in the previous section. A conical projectile localises impact in a smaller zone, leading to more significant failure of layers and enhanced penetration into the composite (see Figure 10) with a higher stiffness plate reduction. 

**Figure 13:**
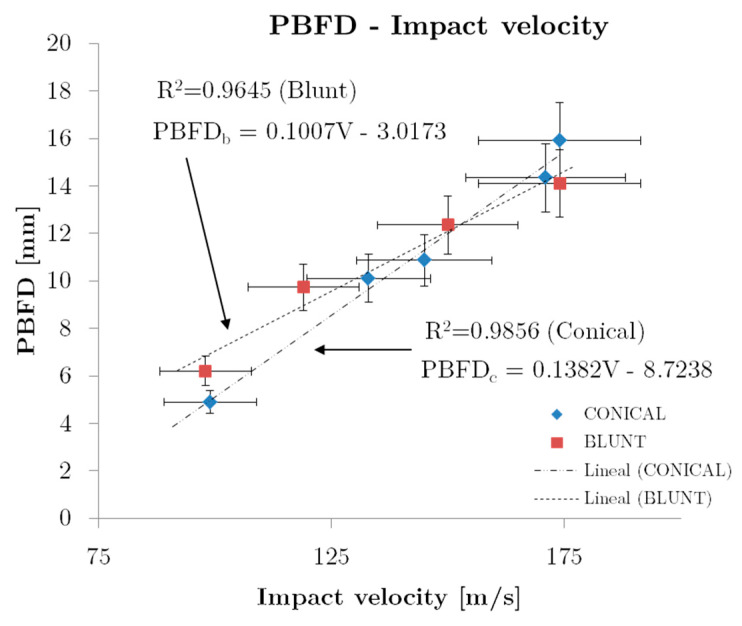
Back Face Deformation—Impact velocity correlation.

A correlation between the permanent back face deformation and the impact velocity for both tip-shapes are shown in Figure 13. A linear dependence of the back-face deformation with the impact velocity for both projectiles can be observed. The correlation coefficients (R-Value) are 0.9645 for blunt and 0.9856 for conical, showing a good correlation of experimental values obtained with linear regression. Although both trends are very similar, the fitting line slope is slightly higher for conical projectiles than blunt ones due to the penetration power of this projectile. 

In the author’s opinion, this correlation for PBFD as a function of the impact velocity in conjunction with the resistance force versus impact velocity as presented in Figure 9, are of great interest to better understand the impact behaviour of aramid composite panels for personal protections in the impact energy range considered.

**Figure 14:**
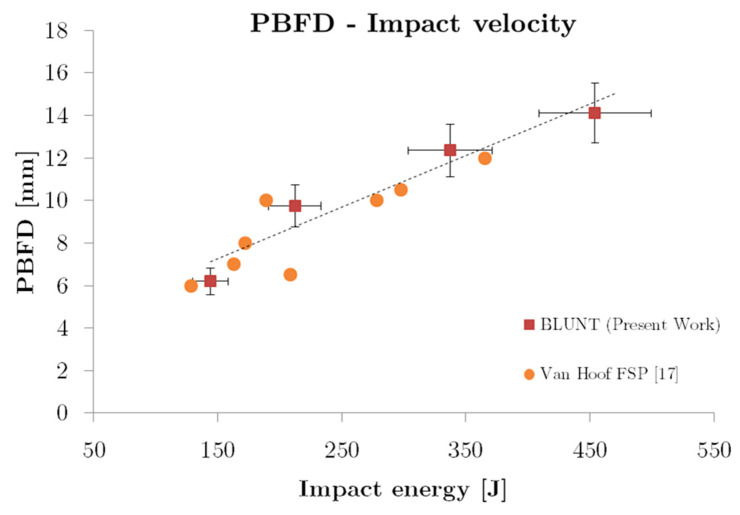
Comparison between existing data on literature and present work.

Although there are other works in the literature about ballistic impact testing on aramid composites, there is a wide variety of impact range (mass, velocity tip-shapes), types of aramid and specimen dimensions that complicate comparisons with the present results. However, within the limited literature for the blunt projectile, the results have been compared with the work developed by Van Hoof [17], see Figure 14. In that work, the dimensions of the specimen, number of composite layers, and the boundary conditions are similar, although the projectile used (FSP) has a reasonably similar geometry to the blunt projectile, the size and the aspect ratio is lower, hence the possible differences that can be observed. Both results present similar trends for the back face deformation (PBFD) in the impact energy range studied, where the PBFD increases linearly in all cases with energy, with a similar slope considering a linear regression.

After this analysis and the content presented in Section 1, it is difficult to compare the results obtained in this study due to the great differences with other works, however, when studying the same material and similar loading situations (impact energy), it provides an idea of the level of deformation when it is subjected to high energy impulsive loads.

## 4. Conclusions

This study has demonstrated the application of different non-destructive image technologies to analyse the final deformation and damage induced in aramid composite plates subjected to ballistic impact. The obtained results, combining computerised tomography (CT) and 3D Scan, revealed a robust methodology for evaluating (qualitatively and quantitatively) defects, damages, deformation in aramid composites, where the use of non-destructive techniques for damage evaluation are not widely dispersed. There is an overall good correlation among both methods used. The setup based on piezoelectric sensors to measure the dynamic force allows relating the impact force to permanent back faced deformation. 

All three technologies can be used to analyse damage or defects in aramid composite materials. However, the only one to estimate the complete damage is the tomography and eventually the 3D scanner if full perforation occurs (e.g., hole). The technique of inspection by force measurement is more complex since a large number of measures are required to obtain the maximum force to relate it to the failure. The main advantages of the use of tomography found in this work are the accuracy of resolution and the ability to obtain internal damage; however, it is a highly expensive and time-consuming method. The 3D scanner technology allows obtaining details of the external geometry (PBFD and/or local failure) in a short time, being appropriate for industrial applications. 

Regarding the differences found between the projectiles used, the CT analysis revealed the greater penetration power of the conical projectile than the blunt projectile for the range of impact velocities used. Note that all the projectiles were made of hard steel and therefore were undeformable. Moreover, the conical projectile produced a greater deformation of the rear face of the plate (PFBD) than the blunt projectile.

This study demonstrates that the advances in computing and sensorisation to analyse the internal damage and external deformation in aramid composites, where typical ultrasonic inspection is not applicable, may have an excellent use for the industry of personal protection such as combat helmets.

## Figures and Tables

**Table 1:** K129 aramid composite used in this work.

Mechanical Properties	K129 Fibre [29]
Young modulus [GPa]	97
Tensile Strength [GPa]	3.45
Elongation at break [%]	3.40
Density [kg/m^3^]	1450

**Table 2:** Microtomography µCT scanning parameters to damage analysis.

Parameter	Data
Current tension	100 kV
Voxel resolution	350 nm/voxel
Image pixel size	50 µm
Nano-focus tube	160 kW
No. of projections	2234
Frame rate	4 images/second

## References

[1] Crouch I.G. (2019). Body armour—New materials, new systems. Def. Technol..

[2] Crouch I.G., Limited W.P. (2017). The Science of Armour Materials.

[3] González-Albuixech V.F., Rodríguez-Millán M., Ito T., Loya J.A., Miguélez M.H. (2019). Numerical analysis for design of bioinspired ceramic modular armors for ballistic protections. Int. J. Damage Mech..

[4] Nayak N., Banerjee A., Datta D. (2012). Ultrasonic assessment of bullet inflicted damage in aramid laminated composites. Def. Sci. J..

[5] Artero-Guerrero J.A., Pernas-Sánchez J., López-Puente J., Varas D. (2015). Experimental study of the impactor mass effect on the low velocity impact of carbon/epoxy woven laminates. Compos. Struct..

[6] Pernas-Sánchez J., Artero-Guerrero J.A., Varas D., López-Puente J. (2014). Experimental analysis of normal and oblique high velocity impacts on carbon/epoxy tape laminates. Compos. Part A Appl. Sci. Manuf..

[7] Artero-Guerrero J.A., Pernas-Sánchez J., Martín-Montal J., Varas D., López-Puente J. (2018). The influence of laminate stacking sequence on ballistic limit using a combined Experimental/FEM/Artificial Neural Networks (ANN) methodology. Compos. Struct..

[8] Pernas-Sánchez J., Artero-Guerrero J.A., Zahr Viñuela J., Varas D., López-Puente J. (2014). Numerical analysis of high velocity impacts on unidirectional laminates. Compos. Struct..

[9] Segreto T., Teti R., Lopresto V. (2018). Non-destructive testing of low-velocity impacted composite material laminates through ultrasonic inspection methods. Characterisations of Some Composite Materials.

[10] Wu L., Wang W., Jiang Q., Xiang C., Lou C.W. (2019). Mechanical Characterization and Impact Damage Assessment of Hybrid Three-Dimensional Five-Directional Composites. Polymers.

[11] Vasudevan A., Senthil Kumaran S., Naresh K., Velmurugan R. (2020). Layer-wise damage prediction in carbon/Kevlar/S-glass/E-glass fibre reinforced epoxy hybrid composites under low-velocity impact loading using advanced 3D computed tomography. Int. J. Crashworthiness..

[12] Fidan S., Sınmazçelik T., Avcu E. (2012). Internal damage investigation of the impacted glass/glass+ aramid fiber reinforced composites by micro-scomputerised tomography. NDT E Int..

[13] Samal S., Kolinova M., Rahier H., Dal Poggetto G., Blanco I. (2019). Investigation of the internal structure of fiber reinforced geopolymer composite under mechanical impact: A micro computed tomography (µCT) study. Appl Sci..

[14] Schilling P.J., Karedla B.R., Tatiparthi A.K., Verges M.A., Herrington P.D. (2005). X-ray computed microtomography of internal damage in fiber reinforced polymer matrix composites. Compos. Sci. Technol..

[15] Karahan M., Jabbar A., Karahan N. (2014). Ballistic impact behavior of the aramid and ultra-high molecular weight polyethylene composites. J. Reinf. Plast. Compos..

[16] Guoqi Z., Goldsmith W., Dharan C.H. (1992). Penetration of laminated Kevlar by projectiles—I. Experimental investigation. Int. J. Solids Struct..

[17] Van Hoof J. (1999). Modelling of Impact Induced Delamination in Composite Materials.

[18] Moure M.M., Rubio I., Aranda-Ruiz J., Loya J.A., Rodríguez-Millán M. (2018). Analysis of impact energy absorption by lightweight aramid structures. Compos. Struct..

[19] Tham C.Y., Tan V.B.C., Lee H.P. (2008). Ballistic impact of a KEVLAR^®^ helmet: Experiment and simulations. Int. J. Impact Eng..

[20] Glossop N.D.W., Tsaw W., Measures R.M., Tennyson R.C. (1989). Image-enhanced backlighting: A new method of NDE for translucent composites. J. Nondestruct. Eval..

[21] Sławski S., Szymiczek M., Kaczmarczyk J., Domin J., Duda S. (2020). Experimental and Numerical Investigation of Striker Shape Influence on the Destruction Image in Multilayered Composite after Low Velocity Impact. Appl. Sci..

[22] Tan L.B., Tse K.M., Lee H.P., Tan V.B.C., Lim S.P. (2012). Performance of an advanced combat helmet with different interior cushioning systems in ballistic impact: Experiments and finite element simulations. Int. J. Impact Eng..

[23] Palta E., Fang H., Weggel D.C. (2018). Finite element analysis of the Advanced Combat Helmet under various ballistic impacts. Int. J. Impact Eng..

[24] Rubio I., Rodríguez-Millán M., Marco M., Olmedo A., Loya J.A. (2019). Ballistic performance of aramid composite combat helmet for protection against small projectiles. Compos. Struct..

[25] Díaz-Álvarez A., Rodríguez-Millán M., Díaz-Álvarez J., Miguélez M.H. (2018). Experimental analysis of drilling induced damage in aramid composites. Compos. Struct..

[26] Rodriguez-Millan M., Tan L.B., Tse K.M., Lee H.P., Miguelez M.H. (2017). Effect of full helmet systems on human head responses under blast loading. Mater. Des..

[27] Rodríguez-Millán M., Ito T., Loya J.A., Olmedo A., Miguélez M.H. (2016). Development of numerical model for ballistic resistance evaluation of combat helmet and experimental validation. Mater. Des..

[28] Rodríguez Millán M., Moreno C.E., Marco M., Santiuste C., Miguélez H. (2015). Numerical analysis of the ballistic behaviour of Kevlar^®^ composite under impact of double-nosed stepped cylindrical projectiles. J. Reinf. Plast. Compos..

[29] Prasad V.V., Talupula S. (2018). A review on reinforcement of basalt and aramid (Kevlar 129) fibers. Mater. Today Proc..

[30] Zhong W.Z., Mbarek I.A., Rusinek A., Bernier R., Jankowiak T., Sutter G. (2016). Development of an experimental setup for dynamic force measurements during impact and perforation, coupling to numerical simulations. Int. J. Impact Eng..

[31] Rodríguez-Martínez J.A., Rusinek A., Pesci R., Zaera R. (2013). Experimental and numerical analysis of the martensitic transformation in AISI 304 steel sheets subjected to perforation by conical and hemispherical projectiles. Int. J. Solids Struct..

[32] Rodríguez-Millán M., Vaz-Romero A., Rusinek A., Rodríguez-Martínez J.A., Arias A. (2014). Experimental Study on the Perforation Process of 5754-H111 and 6082-T6 Aluminium Plates Subjected to Normal Impact by Conical, Hemispherical and Blunt Projectiles. Exp. Mech..

[33] Rodriguez-Millan M., Garcia-Gonzalez D., Rusinek A., Abed F., Arias A. (2018). Perforation mechanics of 2024 aluminium protective plates subjected to impact by different nose shapes of projectiles. Thin-Walled Struct..

[34] Yılmaz E., Gökçen M.G., Demirural A., Baykara T. (2017). Characterization of the damage mechanism of composites against low velocity ballistic impact using computed tomography (CT) techniques. Res. Dev. Mater. Sci..

[35] Min S., Chai Y., Chu Y., Chen X. (2019). Effect of Panel Construction on the Ballistic Performance of Multiply 3D through-the-Thickness Angle-Interlock fabrIc Reinforced Composites. Polymers.

